# Population genetic structure in *Sabatieria* (Nematoda) reveals intermediary gene flow and admixture between distant cold seeps from the Mediterranean Sea

**DOI:** 10.1186/s12862-017-1003-2

**Published:** 2017-07-01

**Authors:** Annelies De Groote, Freija Hauquier, Ann Vanreusel, Sofie Derycke

**Affiliations:** 1 Marine Biology Research Group, Biology Department, Ghent University, Krijgslaan 281, 9000 Ghent, Belgium; 2 Operational Directorate Taxonomy and Phylogeny, Royal Belgian Institute of Natural Sciences (RBINS), Rue Vautier 29, 1000 Brussels, Belgium

## Abstract

**Background:**

There is a general lack of information on the dispersal and genetic structuring for populations of small-sized deep-water taxa, including free-living nematodes which inhabit and dominate the seafloor sediments. This is also true for unique and scattered deep-sea habitats such as cold seeps. Given the limited dispersal capacity of marine nematodes, genetic differentiation between such geographically isolated habitat patches is expected to be high. Against this background, we examined genetic variation in both mitochondrial (COI) and nuclear (18S and 28S ribosomal) DNA markers of 333 individuals of the genus *Sabatieria*, abundantly present in reduced cold-seep sediments. Samples originated from four Eastern Mediterranean cold seeps, separated by hundreds of kilometers, and one seep in the Southeast Atlantic.

**Results:**

Individuals from the Mediterranean and Atlantic were divided into two separate but closely-related species clades. Within the Eastern Mediterranean, all specimens belonged to a single species, but with a strong population genetic structure (Φ_ST_ = 0.149). The haplotype network of COI contained 19 haplotypes with the most abundant haplotype (52% of the specimens) shared between all four seeps. The number of private haplotypes was high (15), but the number of mutations between haplotypes was low (1–8). These results indicate intermediary gene flow among the Mediterranean *Sabatieria* populations with no evidence of long-term barriers to gene flow.

**Conclusions:**

The presence of shared haplotypes and multiple admixture events indicate that *Sabatieria* populations from disjunct cold seeps are not completely isolated, with gene flow most likely facilitated through water current transportation of individuals and/or eggs. Genetic structure and molecular diversity indices are comparable to those of epiphytic shallow-water marine nematodes, while no evidence of sympatric cryptic species was found for the cold-seep *Sabatieria*.

**Electronic supplementary material:**

The online version of this article (doi:10.1186/s12862-017-1003-2) contains supplementary material, which is available to authorized users.

## Background

The deep sea is one of the largest ecosystems on earth, covering two-thirds of the planet and containing a substantial proportion of the earth’s biodiversity. This ecosystem has long been considered a vast and continuous environment with stable biotic, physicochemical and hydrographical conditions [1] which enabled widespread distribution of several deep-sea invertebrate species [2]. However, the deep-sea bottom is also characterized by the presence of fragmented and strongly differentiated ecosystems such as seamounts, trenches, hydrothermal vents and cold seeps (e.g., [3]). The deep sea supports a highly diverse and endemic benthic invertebrate fauna (e.g., [4, 5]). Additionally, molecular evidence suggests the presence of cryptic species complexes (genetically distinct but morphologically identical species; see [4, 6, 7]) of which some are found to be widespread [4, 7, 8] while others appear to be geographically restricted [4].

While our knowledge on the identity, diversity, and distribution of extant species continues to grow, insights on the evolutionary processes that are at the heart of this diversity in the deep sea are lagging behind. Genetic divergence in the marine environment may result from contemporary and historical processes related to the different oceanic basins [9], changes in seafloor topography [10], current regimes [11, 12], depth differences [13, 14], geographic distances [15] and oxygen levels [16]. For instance, genetic divergence in several benthic species is greater between populations at different depths in the same region than between populations of different regions at the same depth (e.g., [14, 17–19]). Also, bathyal organisms generally exhibit much greater population structure than those from the deeper abyss [6, 14, 20].

Next to hydrothermal vents, cold seeps are among the most widely spread deep-sea reducing environments and are characterized by high levels of methane and sulphide, the latter being toxic to most metazoans. Associated fauna therefore need to be adapted to the complexity of the presence of biogenic structures, geochemical conditions and microbial processes. Active seeps have been reported all over the world, even in the Arctic and Antarctic polar regions [21, 22], and from shallow to hadal depths [22, 23]. Since their discovery in the mid-1980s, one of the most puzzling issues has been the influence of past and present connectivity on the nature of cold-seep species assemblages and their geographical distribution [24]. Within the meiofaunal component of cold-seep biota, free-living nematodes are the most prevailing taxon [25].

Nematodes are among the most numerous multicellular taxa on earth, successfully adapted to nearly every ecosystem, but many species are biologically still poorly understood. Different species feed on materials as varied as algae, fungi, small animals, faecal matter and dead organisms [26]. Most nematode species have separate male and female individuals with males using spicules to transfer sperm into the vulva of females. Most free-living nematodes are egg-laying organisms. Yet, a few species, such as *Halomonhystera disjuncta*, are known to be ovoviviparous [27, 28]. The eggs then hatch into larvae, which appear essentially identical to the adults, except for an underdeveloped reproductive system.

Compared to more common deep-sea environments, most cold seeps support highly productive ecosystems characterized by poor nematode species richness, high biomass and elevated dominance of a few genera able to withstand the high concentrations of toxic hydrogen sulphide (e.g., [27, 29–33]). Until now, the genetic structure of nematode species inhabiting different isolated reduced deep-sea systems remained largely unknown (but see [34]). In terms of connectivity and dispersal, nematodes lack a pelagic stage and are therefore considered poor dispersers due to their small size and low swimming ability [35]. However, free-living nematodes and their eggs can become passively suspended and transported in the water column via external forces (e.g., erosion by hydrodynamic forces; [35]), even by very weak bottom currents [36]. Moreover, some nematode species can actively emerge from the sediment and swim in the water column (e.g., [37]). Population genetic studies confirmed restricted nematode dispersal in shallow-water environments at large geographical scales (> 100 km) and revealed cryptic diversity in a wide range of marine nematodes [38–40]. Although some of these cryptic species showed restricted geographical distributions, others were widespread and several of them were distributed sympatrically [40, 41]. Widespread and even cosmopolitan species distributions have been reported in several nematode species [41–44].

Here, we investigate the genetic structure and connectivity in species of heterogeneously distributed cold seeps in the deep sea. We sampled the dominant *Sabatieria* species, *S. mortenseni*, in one cold seep in the Atlantic and four cold seeps in the Eastern Mediterranean Sea to investigate the genetic diversity patterns within and between reduced cold-seep environments.

The genus *Sabatieria* is one of the most common nematode genera in marine benthic habitats, especially in muddy and muddy sand sediments [45], and is one of the dominant deep-dwelling genera in muddy reduced sediments of shallow-water sites all over the world [46–48], including the shelf break and upper slope [25, 49, 50]. The genus can attain high proportions at oxygen minima but gradually disappears almost completely below 2000 m depth [49, 51]. Many of its species are considered eurytopic and tolerant to unstable, highly polluted environments [52, 53]. Currently, 95 *Sabatieria* species are considered valid [54] and are found in different localities around the world (e.g., [55–57]). *Sabatieria* species appear to be divided in five distinct subgroups according to morphological differences related to the type and distribution of precloacal supplements, characteristics of the gubernaculum and apophysis, the number of turns of the amphideal fovea, and the cephalic setae [45]. The specimens investigated in this study belong to the *pulchra* group, which consists of six species, including *S. mortenseni*. Species of this group are distinguished by the disposition of the precloacal supplements, which are usually conspicuous and relatively few in numbers (5–9), and by the characteristic gubernaculum median pieces and short paired cervical setae [45]. *S. mortenseni* has been found in reduced sediments of shallow-water sites in Brazil, USA, Antarctica [58–61] and the Strait of Magellan (Chile) at depths ranging between 8 and 550 m [62]. It is also the dominant species at the REGAB pockmark in the Gulf of Guinea at a depth of more than 3000 m [33]. Based on the particular isolated character of cold seeps and the strong genetic structure in shallow water nematodes, we expected to observe a strong genetic structure between populations of cold seeps located several hundreds of kilometers apart. In addition, based on the presence of substantial cryptic diversity in marine nematodes we also expected to find undiscovered cryptic diversity in these cold seeps.

## Methods

### Sample locations and characteristics

**Fig. 1:**
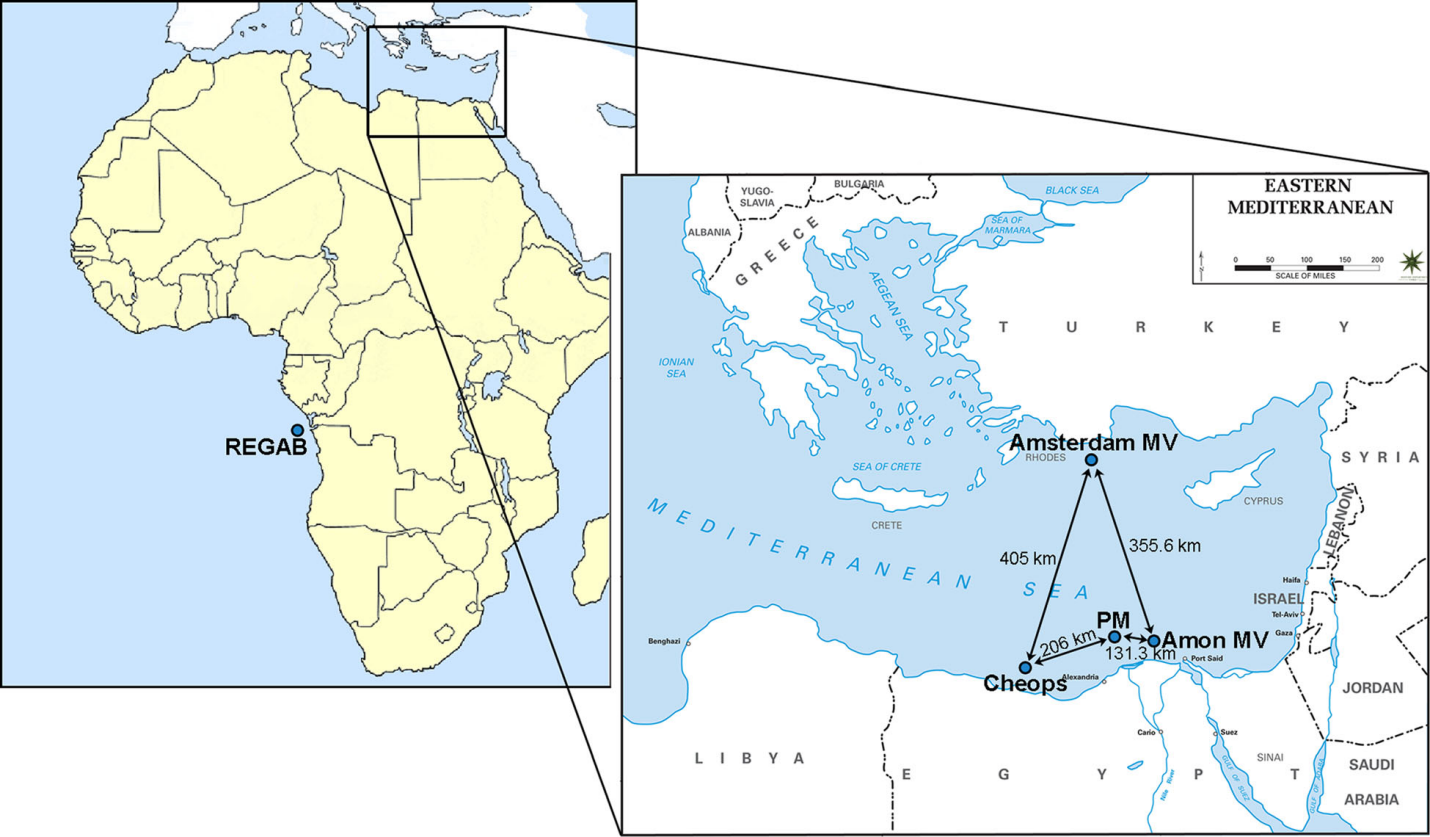
Map of the location of the investigated cold seeps and distances between them. Amsterdam MV, Amon MV, Cheops and PM in the Eastern Mediterranean area; REGAB in the Atlantic Ocean. Source maps: http://www.geography-site.co.uk; http://www.emersonkent.com/map_archive/eastern_mediterranean.htm

*Sabatieria* specimens were collected at five cold seeps, one located in the Atlantic Ocean and four in the Eastern Mediterranean area (Fig. 1). The REGAB cold seep in the Atlantic is located at 3150 m depth on the equatorial West African passive continental margin (Gulf of Guinea; [33]). This 800–1000 m wide and 15–20 m deep pockmark (crater-like depression) [33, 63] is characterized by methane seepage, gas hydrates and the presence of carbonate crusts [64, 65].

The four other cold seeps are located in the Eastern Mediterranean area (Fig. 1) at distances that vary from 131 to 405 km. The most northern cold seep, Amsterdam mud volcano (MV), is located on the southern flanks of the Anaximenes seamount (Anaximander Mountains, Southwestern Turkey; [66]) in the northern Levantine basin. This 2050 m deep MV expels a methane plume of more than 200 m high and harbors methane concentrations of up to 2000 nmol/kg [67]. The other investigated seeps are located at the opposite side of the Eastern Mediterranean sea, at the Nile Deep Sea Fan (NDSF) in the southern Levantine basin. Three major zones of fluid seepage were investigated: the eastern province, the central province (also called the pockmark area) and the Menes caldera. The very active Amon MV, located in the eastern province at ~1118 m depth, is a large volcano of 2.7 km in diameter and 90 m in height [68]. Investigations showed high methane and hydrocarbon emissions (1310 nmol/L) into the water column [69]. In the central province, ship-borne multibeam acoustic images revealed numerous small pockmarks and/or mounds [70, 71]. For this study, the fauna of two major pockmark sites (2A and 2B) and an area close to 2A but located at the lower slope (between ∼ 1650 and 2200 m water depth) were sampled and pooled as PM in the rest of the analyses. Finally, within the Menes caldera, the Cheops MV is an active MV located at 3000 m water depth at the base of the Egyptian continental slope. From the volcanoes flows a mixture of brine, fluids and mud. The brines are expelled through numerous sub-circular vents (a few meters in diameter) which are covered with white sulphidic material produced by sulphate-reducing bacteria [72].

### Sample collection and molecular processing

The cold seeps investigated in this study were the subject of international projects, and were thoroughly investigated during multiple campaigns. Therefore, samples for both morphological and molecular research were available for these particular reduced environments. Samples were collected during MEDECO Leg 2 of RV *Pourqoui Pas?* (November 2007) and MSM13/3 – MSM13/4 campaign of the RV *Maria S. Merian* (November–December 2009) for the Mediterranean seeps, and during the Guineco cruise (2008) onboard RV *Meteor* for the Atlantic REGAB site (Table 1). At each cold seep, multiple cores of reduced sediments were gathered by means of Remotely Operated Vehicles (ROV), sliced per cm and preserved in ethanol or frozen at −20 °C. After extraction of nematodes from the sediment by density gradient centrifugation with Ludox (a colloidal silica polymer; specific gravity 1.18; [73]), *Sabatieria* individuals were identified under a stereomicroscope using diagnostic morphological characteristics. Specimens were randomly picked with a fine needle and put in MilliQ water to remove adhering particles. Prior to molecular analysis, multiple individuals per location (PM(2A): 45; PM(2B): 42; PM: 62; Cheops: 112; Amon MV: 115; Amsterdam MV: 108; REGAB: 34) were photographed using a Leica DMR microscope equipped with a Leica DFC 420 camera to serve as a morphological reference. Individuals examined here were assigned to *Sabatieria mortenseni* based on the number of turns of the amphideal fovea (this study: 3, [60]: 2.5; [61]: 2.5–3; [74]: 2.25–2.5); and for the males, based on the spicule shape, the presence of a sclerotized central part of the apophysis, and the shape of the precloacal supplements. The number of precloacal supplements showed substantial variation in our specimens: males from the Eastern Mediterranean had 6–8 precloacal supplements, and males from REGAB 5–6. The number of precloacal supplements in *S. mortenseni* is 6 according to [45] and 6–7 according to [74]. Multiple characteristics (e.g., amphid diameter, head diameter, anal body diameter) were measured in 18–48 males per cold seep (see Additional file 1: Table S1). Morphometric data were obtained to calculate ratios (e.g., De Man ratio ‘a’, amphid diameter as percentage of the corresponding body diameter) following the instructions found in [45, 74] to assure that specimens from both the Atlantic and Mediterranean area belonged to the same morphological species (see Additional file 1: Table S1). Representative specimens were deposited in the Ghent University Museum, Zoology Collection (voucher codes UGMD104325–104331). Individual nematodes were then immediately transferred in 20 μl Worm Lysis Buffer (WLB: 50 mM KCl, 10 mM Tris pH 8.3, 2.5 mM MgCl_2_, 0.45% NP40, 0.45% Tween20). In this way, 618 *Sabatieria* specimens were collected from the Mediterranean seeps, and 41 from samples of the REGAB seep. DNA was extracted by adding 1.5 μl proteinase K (10 mg/ml) to each WLB-stored specimen, followed by incubation for 1 h at 65 °C and 10 min at 95 °C, and finally centrifugation for 1 min at 14000 rpm prior to usage.

**Table 1:** Overview of the samples used for molecular analysis

Location	Campaign	Date	Latitude (°N)	Longitude (°E)	Depth (m)	Preservation method	n sampled	n processed
PM (2A)	MEDECO-2	08/11/07	32.50021	30.26088	1686	−20 °C	49	10
PM (2B)	MEDECO-2	09/11/07	32.53336	30.35209	1693	−20 °C	50	27
PM	MSM 13/4	10/12/09	32.53317	30.35200	1694	−20 °C	9	0
	MSM 13/4	10/12/09	32.53317	30.35200	1694	−20 °C	69	46
Cheops	MEDECO-2	19/11/07	32.14142	28.16190	3015	−20 °C	127	92
	MEDECO-2	20/11/07	32.14149	28.16196	3015	−20 °C	14	1
Amon	MSM 13/3	07/11/09	32.36747	31.70457	1156	Ethanol	18	9
	MSM 13/3	07/11/09	32.36745	31.70458	1157	−20 °C	60	34
	MSM 13/3	13/11/09	32.36903	31.71091	1120	−20 °C	76	39
Amsterdam	MSM 13/4	25/11/09	35.33467	30.26885	2031	Ethanol	35	12
	MSM 13/4	01/12/09	35.33243	30.26832	2029	−20 °C	111	61
REGAB	M76/3b Guineco	03/08/08	−5.79771	9.71141	3172	−20 °C	41	3

The campaign, date of collection, latitude, longitude and depth (in meters) are given for each core. Also the preservation method, number of *Sabatieria* specimens collected (n sampled) and number of successful sequences (n processed) are mentioned per core

*PM* Pockmark area; *n sampled* number of individuals of *Sabatieria* picked from each core; *n processed* number of individuals yielding a good sequence

For the polymerase chain reaction (PCR), 1 μl of DNA template was used. In this study, 420 base pairs (bp) of the mitochondrial cytochrome *c* oxidase subunit 1 (COI) gene were amplified using the forward primer JB3 (5′-TTTTTTGGGCATCCTGAGGTTTAT-3′) [75] and the reverse primer JB5GED (5′-AGCACCTAAACTTAAAACATARTGRAARTG-3′) [39]. Standard PCR amplifications were conducted in 25 μl volumes for 35 cycles, each consisting of 30 s denaturation at 94 °C, 30 s annealing at 50 °C, and 45 s extension at 72 °C, with an initial denaturation step of 5 min at 94 °C and a final extension step of 10 min at 72 °C. Four μl of each PCR product was loaded on a 1% agarose gel to check the strength and size of the amplified product. After electrophoresis, 420 PCR products, each corresponding to a single nematode specimen, were sent to MacroGen (The Netherlands) for bidirectional sequencing with the primers JB3-JB5GED.

After identifying the different COI haplotypes, DNA from each haplotype was used to amplify two nuclear DNA gene regions: the 18S region of the small ribosomal subunit and the D2D3 region of the conserved 28S large ribosomal subunit. 752 bp of the D2D3 region was amplified with the forward primer D2A (5′-ACAAGTACCGTGAGGGAAAGTTG-3′) and the reverse primer D3B (5′-TCCTCGGAAGGAACCAGCTACTA-3′) [76], while ±950 bp of the 18S region was acquired with the forward primer G18S4 (5′-GCTTGTCTCAAAGATTAAGCC-3′) and the reverse primer 4R (5′-GTATCTGATCGCCKTCGAWC-3′) [77]. PCR conditions were similar to those for COI, except that 40 cycles were applied, and the annealing temperature was augmented to 54 °C. Next to this, an extension time of one minute was applied for the amplification of the 18S gene region. The PCR products of 36 nematodes for 18S and 34 nematodes for D2D3 were sent for sequencing.

### Data analyses

#### Phylogenetic analysis and variation in COI and nuclear markers

COI sequences were checked and trimmed for further phylogenetic analysis in DNASTAR Lasergene Seqman Pro [78]. Primer ends were removed, and sequences which were too short or showed too many uncertain bp were removed from the dataset. The remaining sequences were aligned using CLUSTALW v2 with default parameters as incorporated in MEGA 6.0 [79] and translated using the invertebrate mitochondrial genetic code. A blastx search was performed against the non-redundant protein collection (nr) in GenBank to check the nematode origin of the sequences. Unique haplotypes were identified using a distance matrix based on uncorrected p-distances of untranslated sequences in MEGA. The number of conserved, variable and parsimony informative positions was determined. The best-fit substitution model was selected in jModelTest [80, 81] using the Bayesian Information Criterion (BIC). After that, phylogenetic relationships between haplotypes were constructed in MEGA using Neighbor-Joining (NJ, p-distance model) and Maximum Likelihood (ML, Hasegawa-Kishino-Yano model with gamma distribution as identified by jModelTest; [82]) tree-constructing algorithms, both with 1000 bootstrap replicates. The nematode species *Praeacanthonchus punctatus*, *Ptycholaimellus carinatus* and *Odontophora setosa*, all from the same subclassis (Chromadoria) as *Sabatieria*, were added to the dataset as outgroup (Derycke, unpublished data). The same strategy was followed for the two nuclear markers with the exception that a Tamura-Nei + G substitution model [83] was used in ML tree construction and that outgroups included both unpublished (*Odontophora setosa* and *Praeacanthonchus punctatus*) as well as published sequences extracted from GenBank (*Ptycholaimellus* sp.: accession JN968285-18S; *Praeacanthonchus punctatus*: accession AF210416-D2D3 and *Odontophora* sp.: accession DQ077756-D2D3). For D2D3, there were no *Ptycholaimellus* sequences available. The unique haplotypes found in this study have been submitted to GenBank and are available under accession numbers LT703318-LT703405. Representative DNA samples were deposited in the MoMentuM database of CeMoFe (Centre for Molecular Phylogeny and Evolution), UGent.

#### Population genetic structure in COI

Analysis of Molecular Variance (AMOVA) was performed in ARLEQUIN version 3.5.1.2 [84] and used to analyze the population genetic structure of *Sabatieria* COI sequence variation across the different seeps. Population pairwise Φ_ST_-statistics were calculated (10,000 permutations, 0.05 significance level) to determine which seeps were significantly differentiated from each other. Because the HKY model given by jModelTest is not available in ARLEQUIN, the more inclusive Tamura-Nei (TrN) model [83] with gamma distribution was used to calculate Φ_ST_ and other relevant statistics. Significance levels of the obtained *p*-values were corrected according to the sequential Bonferroni method [85]. Next to this, standard measures of genetic variation within populations, such as nucleotide diversity (π) [86] and haplotype diversity (*h*) [86, 87], and their standard deviation were calculated per cold seep. Tajima’s *D* [88] and Fu’s *Fs* [89] neutrality tests were performed to infer whether sequence evolution was neutral and whether geographical groups showed signs of deviations from neutrality. Significantly negative values for Tajima’s *D* and Fu’s *Fs* reflect an excess of rare polymorphisms in a population, which indicates either positive selection or an increase in population size [90]. These tests were performed both on the haplotype distribution of the separate seeps as well as on the whole dataset as one, with the number of simulated samples set at 1000.

To test whether genetic divergence between populations increased with geographic distance (i.e. isolation-by-distance IBD; [91]), geographic and genetic distances were compared using a Mantel test (1000 permutations; [92]). Both untransformed and log-transformed distances were used to test the IBD model. The geographic distance between populations was measured as the shortest continuous water-surface distance (Fig. 1). To explore the intraspecific relationships between observed haplotypes, a TCS network was constructed with the PopART software (http://popart.otago.ac.nz).

## Results

### Phylogenetic analysis of COI

**Fig. 2:**
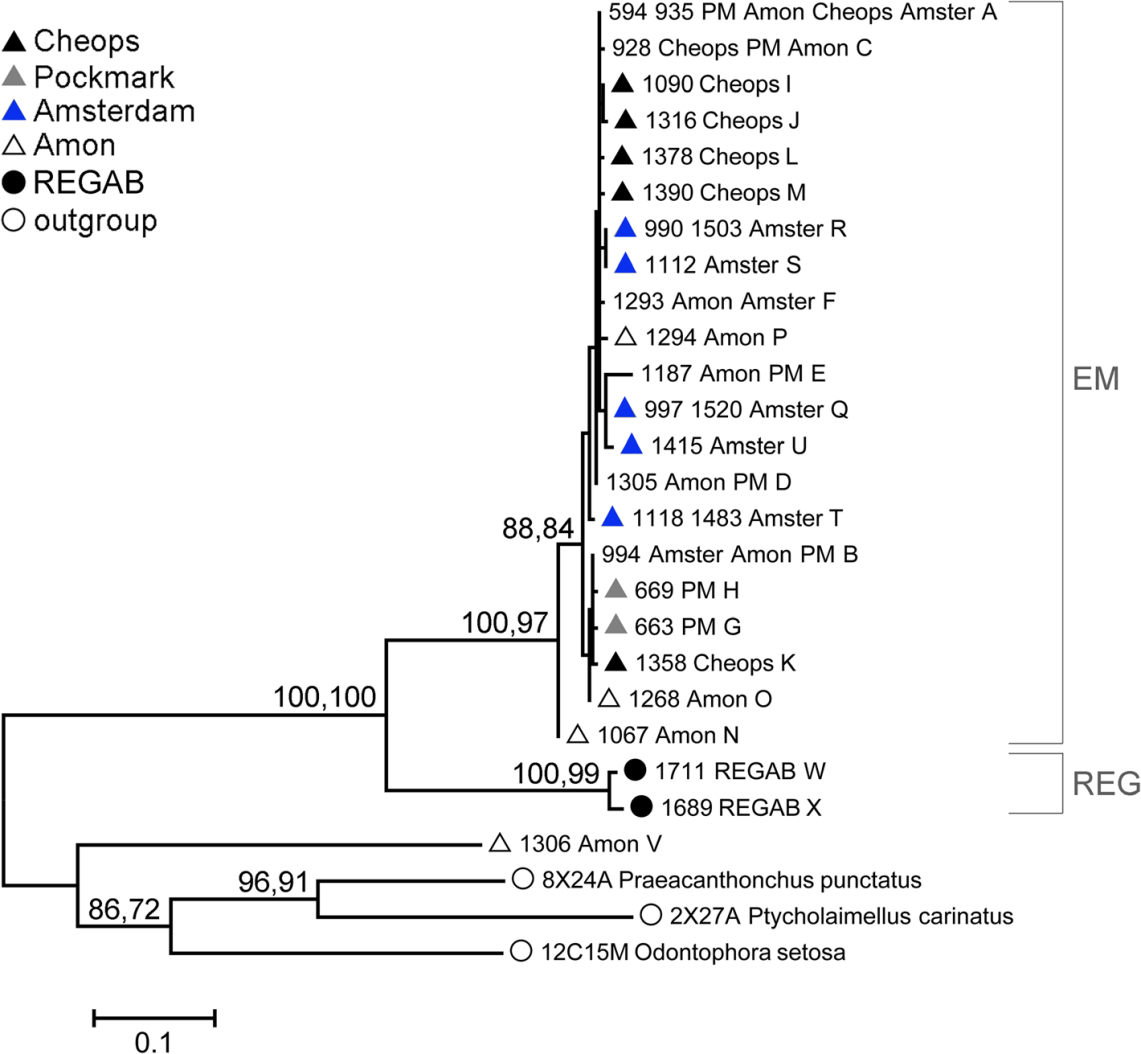
Phylogenetic ML tree of the COI haplotype sequences with indication of two clades. EM = Eastern Mediterranean clade; REG = REGAB clade. Values along branches are bootstrap values from the NJ algorithm based on p-distance and the ML procedure based on HKY + G, separated by a comma. Bootstrap values lower than 70 are not visualized in the figure. Each organism received a unique identification code (PCR code_Cold seep(s)_haplotype). In case haplotypes occurred at only one geographical location, their origin is indicated by a symbol and color (see legend). Haplotypes shared among several locations are not marked

Sequence alignment yielded a COI fragment of 420 bp long. This fragment contained 135 conserved and 264 variable positions, 193 of which were parsimony informative and 69 of which were singletons. Both the NJ and ML tree topology of COI haplotypes showed two well-supported clades (Fig. 2). The REG clade contained the two sequences from the REGAB cold seep (Table 1), while the EM clade included all 331 sequences from the Eastern Mediterranean cold seeps, except haplotype V. This latter sequence was highly divergent from the other sequences (p-distance EM: 0.400–0.408; REG: 0.444). P-distances between the two clades ranged between 0.234–0.269, while p-distances within clades were much lower (EM: 0.003–0.043; REG: 0.015). No subdivision according to geographic location could be discerned within the EM clade.

### Phylogenetic analysis of the nuclear markers

**Fig. 3:**
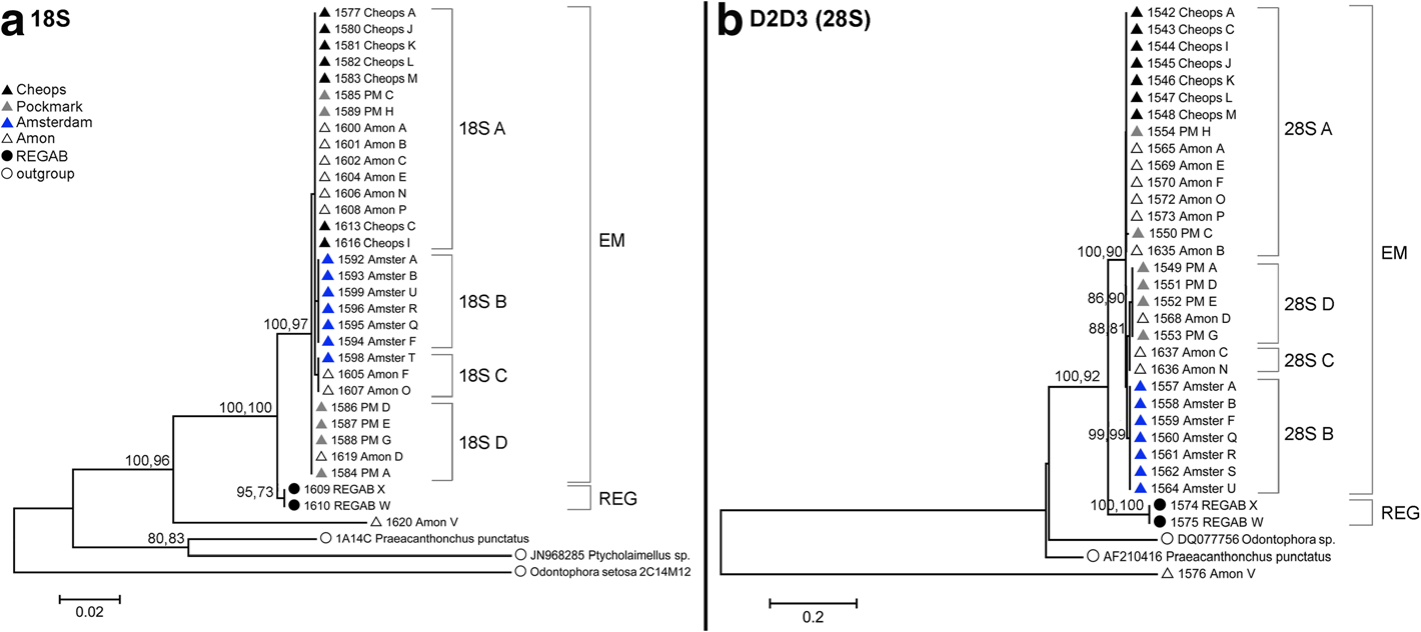
Phylogenetic ML trees for (**a**) 18S and (**b**) the D2D3 marker of 28S. EM: Eastern Mediterranean clade; REG: REGAB clade. Values along branches are bootstrap values of both NJ (p-distance) and ML (TrN + G) procedures, separated by a comma. Bootstrap values lower than 70 are not visualized in the figure. Each organism received a unique identification code (PCR code_Cold seep_haplotype). Symbols and colors indicate the origin of the sequences

The 18S fragment was 942 bp long, contained 678 conserved and 262 variable positions, 98 of which were parsimony informative and 163 of which were singletons. Two insertions of a single bp were noted in two outgroup species (*Praeacanthoncus punctatus* and *Ptycholaimellus* sp*.*). The D2D3 fragment was 752 bp long with 206 conservative and 531 variable positions, of which 155 parsimony informative and 373 singletons. The NJ and ML analysis of both markers showed congruent tree topologies which were similar to the topology of the COI tree, with two well-supported clades (Fig. 3). P-distances between the two clades were 0.012–0.014 for 18S, and 0.120–0.172 for D2D3. As for COI, p-distances within clades were much lower (18S: EM: 0.000–0.002; REG: 0.000; D2D3: EM: 0.000–0.019, REG: 0.000). There was no clear subdivision according to geographic location in the nuclear markers. Only the sequences from the Amsterdam MV formed a separate group with high bootstrap support in the tree of the D2D3 fragment (Fig. 3b, group 28S B). Similar to COI, haplotype V was clearly separated from all other *Sabatieria* sequences with high p-distance values (18S: EM: 0.082–0.090, REG: 0.080; D2D3: EM: 0.627–0.643, REG: 0.631–0.636). A Blast search of its 18S sequence identified it as *Terschellingia longicaudata* (95–98% similarity), a species that is abundantly present at Amon MV. This haplotype was therefore excluded from further population genetic analyses.

Similarly, since 5% COI sequence divergence is commonly observed between different nematode species [37], and because of the congruence between mitochondrial and nuclear markers, the REG clade was considered as an independently evolving lineage, and was omitted from the dataset to investigate population genetic structure within the Eastern Mediterranean clade.

### Population genetic structure of COI

**Fig. 4:**
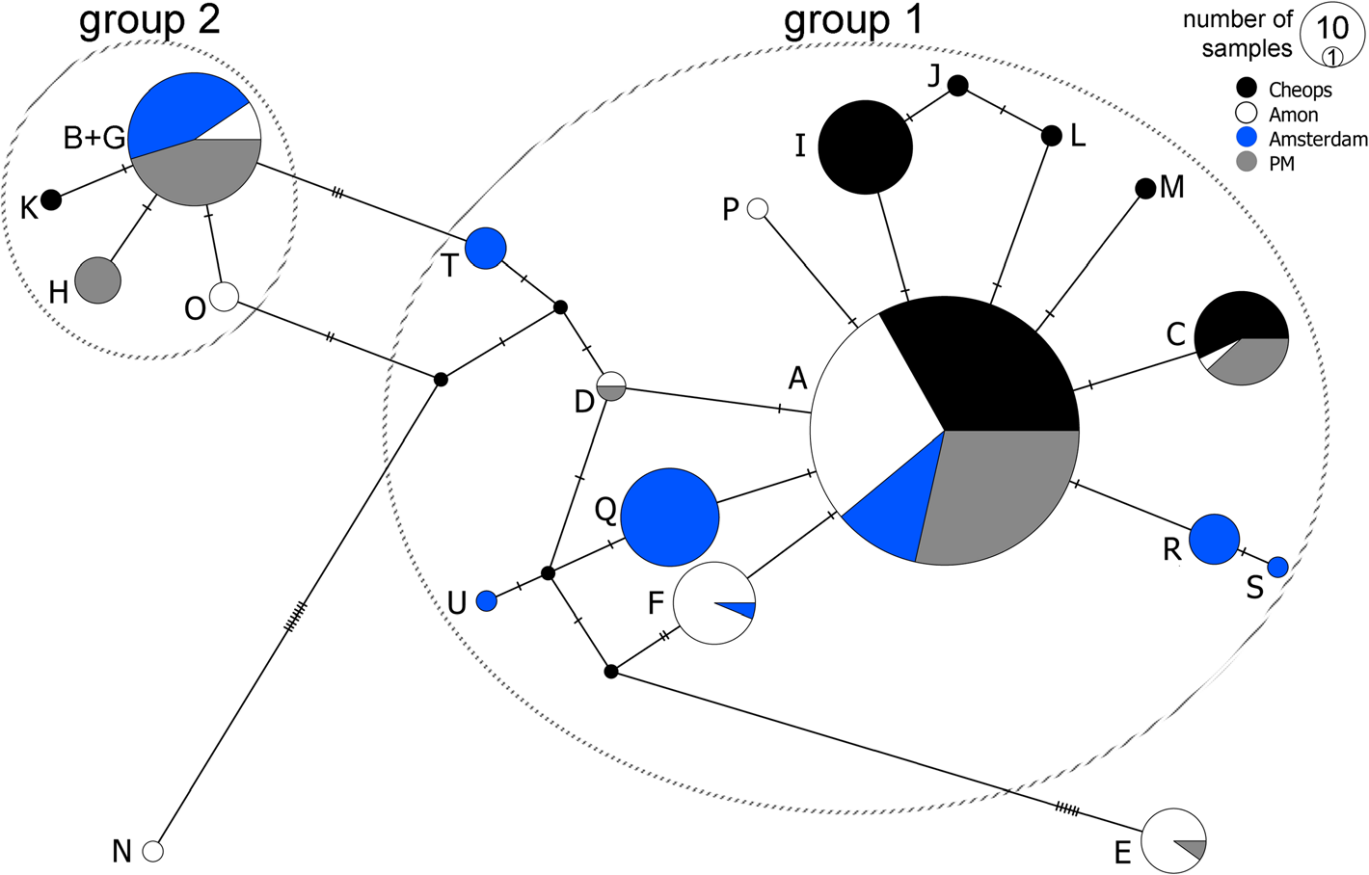
TCS network constructed with the haplotypes found in the EM clade. Each circle represents a haplotype, while the different colors represent the cold seeps. The division and size of the circles shows the frequency of this haplotype in each cold seep. Hatch marks along connecting branches indicate the number of substitutions between two haplotypes. Small black circles represent missing haplotypes

A total of 331 individuals were included in the population genetic analyses (only EM clade sequences), divided evenly over all four cold seeps from the Eastern Mediterranean area (Table 2). A fragment of 393 bp long was obtained, containing 33 variable positions, 15 parsimony informative and 18 singletons. In total, 21 haplotypes were found. The number of haplotypes per seep was comparable, with seven haplotypes found at Cheops and PM, eight at Amsterdam MV, and nine at Amon MV. At each seep, two or three haplotypes were found regularly, while the remaining haplotypes were rare (Table 2). Haplotype A was the most widely distributed and abundant haplotype, occurring in all cold seeps and in densities of at least twice that of the second most abundant haplotype. The only exception was Amsterdam MV, where haplotype A (18 times) was slightly less common than Q (23 times) and B (19 times). Next to haplotype A, five other haplotypes (B-F) were also found in multiple seeps. All other haplotypes were unique to one seep, or even to a single specimen (Table 2, Fig. 4).

**Table 2:** Overview of the frequency of the different haplotypes found in Eastern Mediterranean cold seeps

	A	B	C	D	E	F	G	H	I	J	K	L	M	N	O	P	Q	R	S	T	U	n	*h*	π
PM	49	1	8	1	1	-	18	5	-	-	-	-	-	-	-	-	-	-	-	-	-	83	0.598 ± 0.049	0.011 ± 0.006
Cheops	56	-	12	-	-	-	-	-	21	1	1	1	1	-	-	-	-	-	-	-	-	93	0.576 ± 0.045	0.002 ± 0.002
Amon	48	4	1	1	9	15	-	-	-	-	-	-	-	1	2	1	-	-	-	-	-	82	0.616 ± 0.052	0.010 ± 0.006
Amsterdam	18	19	-	-	-	1	-	-	-	-	-	-	-	-	-	-	23	6	1	4	1	73	0.773 ± 0.022	0.012 ± 0.007
Total	171	24	21	2	10	16	18	5	21	1	1	1	1	1	2	1	23	6	1	4	1	331		

The total number of specimens, the haplotype diversity *h* and nucleotide diversity π per cold seep are stated

*h* = haplotype diversity; π = nucleotide diversity

The TCS network further indicated the dominance of haplotype A, which was found in all four seeps (Fig. 4). Two groups of haplotypes differing in only one mutation with the closest related haplotype could be identified, and both groups had haplotypes in all four cold seeps (group 1 and group 2 in Fig. 4). Group 1 contained 14 haplotypes, of which 5 were only found in Amsterdam MV, 4 only in Cheops and 1 only in Amon. Group 2 contained 5 haplotypes, with Cheops, PM and Amon each having one private haplotype. In addition to these two groups of haplotypes, haplotypes E and N showed 6 and 8 mutational differences with the closest (missing) haplotype.

Haplotype diversity (*h*) was higher in Amsterdam MV (0.773 ± 0.022) than in the other seeps (between 0.576 ± 0.045 and 0.616 ± 0.052), while nucleotide diversity (π) was an order of magnitude lower in Cheops than in the three other seeps (Table 2). AMOVA resulted in a high Φ_ST_-value (0.149, *p* < 0.001), attributing 15% of the genetic variability in the dataset to differences between locations (Table 3). All pairwise Φ_ST_-values were high (0.111–0.283) and significantly different from zero after Bonferroni correction (Table 4). The mantel test did not show any significant correlation between genetic and geographic distances, for both untransformed (*p* = 0.390, correlation coefficient = 0.240) and log-transformed (*p* = 0.459, correlation coefficient = 0.204) distances. The same was true for the three seeps in the southern part of the East Mediterranean (Cheops, PM and Amon) when analyzed separately (*p* = 0.4995, correlation coefficient = 0.363). Tajima’s *D* was only significantly negative for Cheops (*D* = −1.649, *p* = 0.025). None of Fu’s *Fs* were significantly different from zero. Also the analysis of the whole dataset as one group did not yield significant results (Tajima’s *D* = −0.819, *p* = 0.213; Fu’s *Fs* = −1.672, *p* = 0.371).

**Table 3:** Results of the AMOVA analysis for the COI marker

Source of variation	df	Sum of squares	Variance components	% of variation
Among populations	3	76.686	0.28966 Va	14.92
Within populations	327	540.002	1.65138 Vb	85.08
Total	330	616.688	1.94104	
*Φ*_ST_ = 0.149	*p*-value <0.001			

The four Eastern Mediterranean seeps are treated as separate populations *df* = degrees of freedom

**Table 4:** AMOVA pairwise Φ_st_-values for COI

	PM	Cheops	Amon	Amsterdam
PM				
Cheops	0.232			
Amon	0.111	0.129		
Amsterdam	0.061	0.283	0.119	

All values are significant after Bonferroni correction (*p* < 0.05)

## Discussion

Our results show that *Sabatieria mortenseni* is present in all samples in cold seeps from the East Mediterranean. The continuous character and the presence of currents as a large-scale transportation mechanism of organisms are likely to facilitate dispersal and gene flow in the marine environment and may result in wide species distributions (e.g., deep-sea foraminifera in Arctic and Antarctic sediments [93, 94]; see also [2]). Also for nematodes, widespread distributions have been reported. For example, the nematode *Litoditis marina* maintains trans-Atlantic populations, and specimens have been found floating in macroalgae rafts in the North Sea [41]. Bik and co-authors [42] found evidence for cosmopolitan marine Oncholaimid species with identical gene sequences (18S, 28S and COI) at trans-Atlantic sample sites. Bhadury and co-workers [43] revealed that the majority of the morphologically defined *Terschellingia longicaudata* specimens shared a single 18S rRNA sequence and apparently belonged to a single species distributed from the British Isles to Malaysia in a wide range of marine habitats and ecological ranges. The high similarity of the *T. longicaudata* from Amon MV in this study with the shallow-water specimens described in [43] (98% similarity; Accession No.: AM941225, AM941226 and AM234716) is indicative of a wide distribution for this species. Nematodes can become passively suspended and transported in the water column by currents [35, 36]. This might not only apply to the organism itself, but also to the eggs, although this latter possibility has not been investigated yet. Next to passive transport, some nematode species can actively swim in the water column, with widespread species distributions as a result [41–43].

For *Sabatieria*, community analysis of the cold-seep environments analyzed in this study revealed that the genus (with *S. mortenseni*) was abundantly present in all reduced sediments sampled (relative abundances 10.6–88.6%; personal observation). The species was also present in the surrounding oxidized sediments, albeit in much lower abundances (0.0–6.4%; personal observation), indicating its preference for hypoxic or anoxic conditions. These data suggest a widespread distribution in the area under study. However, the extent of genetic similarity between *Sabatieria mortenseni* from the non-reduced environment and the cold seeps is not clear, since specimens from the non-reduced environment were preserved in formalin.

### Genetic structure of *Sabatieria mortenseni* shows intermediate gene flow between cold seeps

The strong genetic structure revealed by AMOVA and the high pairwise Φ_st_ values suggest that suspension and transport by currents are not enough to establish extensive gene flow between the *Sabatieria* populations of the different cold seeps. Instead, several haplotypes are shared between seeps, the number of mutations between haplotypes is low and private haplotypes are present in each cold seep, all indicating that intermediate gene flow is occurring without long-term barriers to dispersal between cold seeps (cf. pattern V in [95]). The presence of *S. mortenseni* in the surrounding environment may indicate that the distribution of this species in the deep sea is much more homogeneous than anticipated. Evidently, widely distributed species that are not restricted to certain habitat types may have a larger capacity to disperse than narrowly distributed, specialized species. Even though the abundances of *S. mortenseni* in the surrounding sediment are low, such small local units may form part of a source-sink system facilitating gene flow between distant populations.

The East Mediterranean is one of two sub-basins of the Mediterranean Sea and hosts a complex array of current systems, including deep-water formation in the Adriatic Sea (Eastern Mediterranean Deep Water EMDW; [96]), cyclonic and anticyclonic gyres, and small energetic eddies [97, 98]. Main deep-water currents flow from west to east, along the North African coastline in the southern Ionian and Levantine basin (passing the Nile Deep Sea Fan, i.e. location of Cheops, PM and Amon MV) towards the northern Levantine basin (i.e. location of Amsterdam MV) [99]. Therefore, physical connections between the different cold seep environments of this study are theoretically possible and can explain the presence of several shared haplotypes between the cold seeps.

Even in the presence of currents able to mediate long-distance dispersal of organisms and eggs in relatively homogeneous habitats, dispersal success tends to decrease with increasing distance, resulting in larger genetic dissimilarity between populations that are more distant from each other (i.e. stepping-stone model; [91]). Given the scattered distribution of deep-sea cold seeps and hydrothermal vents, such distance effects have frequently been invoked to explain genetic divergence in chemosynthetic invertebrate species with pelagic larvae [100–103]. For the passively dispersing nematodes which lack such a larval stage, the isolation-by-distance effect might be even more pronounced. However, no significant isolation-by-distance for the *Sabatieria* populations of the East Mediterranean cold seeps was observed (see Mantel test results). This was also evident from the COI phylogenetic tree where populations did not cluster according to their geographic location (Fig. 2). Instead, genetic distances between closely located seeps (e.g., Amon MV and PM) were sometimes larger than between seeps further apart (e.g., Amsterdam MV and PM; Table 4). Such a pattern where genetic structuring does not correlate with geographic distance (i.e. chaotic genetic patchiness) is quite common in the marine environment for organisms with larval development [104–108], but also for nematodes [109], and can be explained by the multidirectional movement of organisms due to turbulent and nonlinear water currents, as well as by other ecological factors (e.g., variable local natural selection on settling larvae; [105]).

### Few mutations between haplotypes point to the absence of long-term geographic barriers between cold seeps

Besides contemporary physical drivers for genetic population differentiation (currents, distance), historical events often leave their mark on current species distributions. On an evolutionary timescale, the Mediterranean deep-sea environment and its fauna are relatively young compared to that of other oceans. Our results show a significant population genetic structuring between the cold seeps within the Eastern Mediterranean sea which may reflect past isolation and (re-) colonization events. The haplotype network for COI consisted of a few central widespread and abundant haplotypes (A and B) surrounded by multiple private haplotypes (Fig. 4, group 1 and 2). Such a pattern is characteristic for population expansion, but statistical confirmation of such an event was limited: Tajima’s *D* was only significantly negative for Cheops MV. Instead, the two haplotype groups were separated by 2 or 3 mutations suggesting recent admixture between previously isolated populations (see also [95, 110]). The higher number of haplotypes in group 1 and the high abundance of the central haplotype A in all cold seeps indicate that this cluster of haplotypes has a longer evolutionary history in the four cold seeps than haplotypes from group 2, which was much less abundant and diverse than group 1 (Fig. 4). The high number of mutations in haplotypes E and N, their very low abundance and their restriction to the Amon cold seep (and PM for E) in the south suggest that individuals harboring these haplotypes most likely originated in locations outside our sampling area, in the southern part of the Mediterranean. In this scenario, the Amon cold seep has been colonized by four independent events (one event containing haplotypes from group 1, one containing haplotypes from group 2, one containing haplotype N and one containing haplotype E), whereas the other cold seeps were colonized by two (Cheops and Amsterdam MV) or three (PM) separate events. Overall, haplotype and nucleotide diversity for *Sabatieria* populations were similar to values reported for shallow-water nematodes (e.g., [38, 111]) and generally low (especially nucleotide diversity).

Finally, the genetic structure present in *S. mortenseni* may result from a strong adaptation to the particular local conditions at the different cold seep habitats. Such a scenario of differentiation by adaptation seems plausible considering the environmental heterogeneity typically found in chemosynthetic habitats [112]. In this case, local selective pressures could prevent the successful exchange of individuals (e.g., [113]), leading to isolation of the gene pools of the different populations. However, based on the data at hand it is difficult to assess whether such a mechanism can be invoked in the case of *Sabatieria* in the East Mediterranean.

### Cryptic speciation

Our results show the presence of cryptic species within *Sabatieria mortenseni* from seeps located in different ocean basins: genetic evidence pointed towards two independently evolving lineages, one in the Eastern Mediterranean and one in the Southeast Atlantic region. Yet, no differences in diagnostic morphological features were observed and morphometric characters overlapped with those of *S. mortenseni* reported in [45, 74] (Additional file 1: Table S1). In general, cryptic species are common in the marine environment (e.g., [114]). Numerous cryptic species exist among the vesicomyid clams [115, 116] and lepetodrilid limpets [117] that dominate vents and seeps worldwide. Cryptic species have also frequently been detected in free-living marine nematodes from intertidal environments with sympatric distributions [38, 39, 118]. In contrast, populations of the marine nematode *Paracanthonchus gynodiporata* living on macroalgae were genetically homogeneous across more than 1000 km Brazilian coastline but showed substantial phenotypic plasticity [44]. Cryptic species have also been reported in deep-sea nematodes: *Halomonhystera disjuncta* from the Håkon Mosby mud volcano is genetically distinct from the intertidal *Halomonhystera disjuncta* [34] and two cryptic species have recently been discovered in *Sabatieria* from the continental shelf in Antarctica [119]. The current study shows that cryptic diversity is also present in nematodes dominating cold seeps in the deep-sea floor, but apparently only at a larger geographic scale. Although morphometric characters overlapped with *S. mortenseni* from [45, 74], specimens from the REGAB cold seep have a larger body size than *S. mortenseni* reported from the literature (Additional file 1: Table S1). Yet, the body size of the REGAB specimens overlaps with that of specimens from Amon and Amsterdam. We measured a large number of males (18–28 per cold seep in the Mediterranean Sea) and find a much larger morphological plasticity in *S. mortenseni* from the cold seeps than observed in *S. mortenseni* from the subtidal area near Rio de Janeiro [74]. Unfortunately, no sequence data are available from specimens from Rio de Janeiro to verify the extent of genetic differentiation between *S. mortenseni* from both areas.

Surprisingly, there was no evidence for cryptic speciation within the East Mediterranean despite the fact that geographical distances were much larger than those in previous studies in marine nematodes [109]. Because of the young age of the Mediterranean Sea (5.3–2 Myr), speciation of the *Sabatieria* populations may not have been completed yet. As a reference, cryptic species found in the shallow-water nematode *Litoditis marina* are at least 6.5 Myr old [120]. Nevertheless, the high variability of local cold-seep conditions (e.g., heterogeneity in fluid composition of sulphide and methane; see Methods section) and low exchange between cold seeps could induce perfect conditions for ecological speciation. We therefore expected cryptic speciation in disjunct cold-seep areas. However, *Sabatieria* species are known to withstand fluctuations in environmental chemistry as they regularly are the dominant genus in various reduced ecosystems (e.g., [27, 30–33]). The lack of cryptic species in these Mediterranean cold seeps can thus be due to the fact that this species, after spreading in the Mediterranean area, still shows sufficient gene flow to homogenize gene pools.

## Conclusion

The populations of the marine nematode *Sabatieria mortenseni* inhabiting cold seeps from the bathyal East Mediterranean Sea and Atlantic Ocean are genetically structured. The presence of shared haplotypes and the low number of mutations between haplotypes indicate that barriers to dispersal between these cold seep populations are relatively recent. The low abundances of *S. mortenseni* in the surrounding sediment together with water currents in the area may form part of a source-sink system facilitating gene flow between distant populations. Our results further show the presence of cryptic species within *S. mortenseni* from seeps located in different ocean basins: genetic evidence pointed towards two independently evolving lineages, one in the Eastern Mediterranean and one in the Southeast Atlantic region but no differences in diagnostic morphological features were observed. Our results expand our knowledge on genetic diversity in marine nematodes and confirm that a single nematode species can be widely distributed despite the lack of pelagic larval stages.

## Additional file

Additional file 1: Table S1. Morphometric analysis of *S. mortenseni*. (DOC 44 kb)
