# Supplementary material for: Population genetic structure in Sabatieria (Nematoda) reveals intermediary gene flow and admixture between distant cold seeps from the Mediterranean Sea
Source: BMC Evol Biol. 2017 Jul 1;17:154. doi: 10.1186/s12862-017-1003-2 (PMC5494145; doi:10.1186/s12862-017-1003-2)
Supplement: Additional file 1: Table S1. — Morphometric analysis of S. mortenseni. (DOC 44 kb) [file 12862_2017_1003_MOESM1_ESM.doc]

**Population genetic structure in *Sabatieria* (Nematoda) reveals intermediary gene flow and admixture between distant cold seeps from the Mediterranean Sea**

De Groote Annelies^1^, Hauquier Freija^1^, Vanreusel Ann^1*^, Derycke Sofie^1,2^

^1^ Marine Biology Research Group, Biology Department, Ghent University, Krijgslaan 281, 9000 Ghent, Belgium, degroote.annelies7@gmail.com, freija.hauquier@ugent.be, ann.vanreusel@ugent.be

^2^ Operational Directorate Taxonomy and Phylogeny, Royal Belgian Institute of Natural Sciences (RBINS), Rue Vautier 29, 1000 Brussels, Belgium, sofie.derycke@naturalsciences.be

**BMC Evolutionary Biology**

SUPPORTING INFORMATION

**Table S1. Comparison between multiple ratios from male *Sabatieria* individuals from the cold seeps investigated in this study, and the ratios found for *S. pulchra*, *S. punctata* and *S. mortenseni* [45], and *S. mortenseni* [74].** L: total body length (µm), a: De Man ratio (total body length/maximum body diameter), A%: amphid diameter as percentage of corresponding body diameter, Ps: number of precloacal supplements, T: tail length measured in cloacal body diameters, n: number of male individuals measured.

|  | **L** | **a** | **A%** | **Ps** | **T** | **n** |
| --- | --- | --- | --- | --- | --- | --- |
| Amsterdam | 1511.7 – 2328.6 | 25.2 – 44.9 | 37.4 – 73.6 | 6 – 8 | 3.2 – 4.7 | 18 |
| Amon | 1908.3 – 2310.8 | 36.7 – 44.6 | 56.7 – 67.3 | 6 – 8 | 3.1 – 4.6 | 19 |
| PM | 1601.1 – 1960.3 | 23.6 – 43.7 | 53.1 – 67.2 | 6 – 8 | 2.0 – 4.4 | 48 |
| Cheops | 1612.1 – 1975.6 | 30.0 – 44.3 | 51.5 – 58.4 | 6 – 8 | 3.3 – 4.3 | 22 |
| REGAB | 2137.8 – 2850.5 | 30.9 – 52.9 | 53.9 – 72.4 | 5 – 6 | 3.1 – 5.2 | 34 |
| **Platt (1985)** |  |  |  |  |  |  |
| *S. pulchra* | 1270 – 2500 | 28 – 45 | 55 – 67 | 6 – 9 | 3.3 – 3.4 |  |
| *S. punctata* | 990 – 1350 | 33 – 38 | 70 – 75 | 5 – 8 | 3.2 – 3.9 |  |
| *S. mortenseni* | 1900 | 47 | 80 | 6 | 3.2 |  |
| **Botelho (2014)** |  |  |  |  |  |  |
| *S. mortenseni* | 1323 – 1512 | 31.5 – 37.8 | 61.5 – 85.7 | 6 – 7 | 3.4 – 3.8 |  |
